# Expression of α4, αv, β1 and β3 integrins during the implantation window on blastocyst of a mouse model of polycystic ovarian syndromes

**Published:** 2014-09

**Authors:** Fatemeh Peyghambari, Saeid Amanpour, Mehri Fayazi, Mahnaz Haddadi, Samad Muhammadnejad, Ahad Muhammadnejad, Mehdi Salimi, Zohreh Mazaheri

**Affiliations:** 1 *Department of Anatomical Sciences, Faculty of Medical Sciences, Islamic Azad University, Yazd Branch, Yazd, Iran.*; 2 *Vali-e-Asr Reproductive Health Research Centre, Tehran University of Medical Sciences, Tehran, Iran.*; 3 *Department of Anatomical sciences, Medical Sciences Faculty, Tarbiat Modares University, Tehran, Iran.*; 4 *Tumor Model Research Center, Cancer Institute of Iran, Tehran University of Medical Sciences, Tehran, Iran.*; 5 *Department of Physiology, Medical Sciences Faculty, Tarbiat Modares University, Tehran, Iran.*

**Keywords:** *Integrin*, *Blastocyst*, *Implantation*, *Polycystic ovarian syndromes*

## Abstract

**Background:** It has been hypothesized that blastocyst integrin expression changes can affect the spontaneous miscarriage in polycystic ovarian syndromes (PCOS).

**Objective:** In this study, the profile of integrin genes and proteins was investigated on blastocyst of the PCOS experimental mouse model.

**Materials and Methods: **30 NMRI female mice were equally divided into 3 groups: control, experimental [PCOS that was injected estradiol valerate (40 mg/kg)]. After 8 weeks, each group was hyper stimulated by PMSG and HCG. Vaginal plaque was checked, and mice were investigated 5 days after the test. Progesterone and estradiol levels were determined; α4, αv, β1 and β3 integrin genes and protein of blastocysts were examined by real time PCR method and immunohistochemistry, respectively.

**Results: **Estradiol level was significantly increased (p≤0.035) in PCOS group. Based on our finding, the ratio of genes' expressions αv, β3, β1 and α4 in PCOS to control group was 0.479±0.01, 0.5±0.001, 2.7±0.4 and 1.023±0.2 respectively. Genes expression showed a great difference (p≤0.001) between β3, β1 and αv in PCOS compared to other groups. αv and β3 integrin proteins expressed in all groups but intensity of these proteins in PCOS groups, was lower than other groups.

**Conclusion:** Pattern of αv and β3 integrins expression on the mouse blastocyst surface has an important effect during the implantation window. This pattern has changed in PCOS model and might have a great influence on implantation failure. Therefore, this experimental study suggests that a great attention to this problem may be essential in patients who are involved.

## Introduction

Polycystic ovary syndrome (PCOS) was first explained by Stein and Leventhal in 1935, distinguished by the association between polycystic ovaries and chronic anovulation, obesity, and hyperinsulinemia, amenorrhea, infertility, and hirsutism in human (1). It is estimated that it affects approximately 5-7% of women at reproductive age, characterizing the most frequent reason of an ovulatory infertility (2, 3). This proposes that anovulation is not the only cause of infertility, means that endometrial receptivity might have a critical role in the concern and development of pregnancy in women with PCOS (2, 4). 

The blastocyst implantation seems to be the major limiting factor for the establishment of pregnancy in a large number of gynecological diseases, including PCOS, and treatment to develop implantation rates are likely to be taken to this course (5). Implantation is preceded by a close interaction of embryonic trophoblast and endometrial epithelial cells that is known as adhesion or attachment. Blastocyst implantation in mice and humans depends on the molecular cross talk of differentiated, adhesion-competent trophoblast cells with extracellular matrix (ECM) components of the receptive uterus, matrix-degrading enzymes and their inhibitors, growth factors, cytokines, and angiogenic peptides (6-10). 

Each of these factors, when expressed or inhibited at the exact time, may contribute to endometrial receptivity or no receptivity. Changes in the expression or distribution of integrin receptors on the trophoblast cells may develop the adhesive and migratory behavior of the trophoblasts (11-13). Integrin subunits α and β have been demonstrated to be expressed in trophoblast cells of preimplantation and implanting mice embryos (14). Abnormal expression of α4, αv, and β1 revealed to be related to implantation failure and abnormal placental development in mice and human (15). 

A variable profile of expression of certain integrins in the murine uterus during the reproductive cycle and pregnancy proposes that these proteins may be directly involved in the process of implantation and in normal placental formation and function (13). Abnormal expressions of α4 and β1 integrin in mice have been related to certain unexplained cases of infertility in human and the regulation of integrin expression during blastocyst activation and implantation were not clear (16, 17). It is clear that these integrin molecules have various expression patterns during the implantation window in uterus and blastocyst surface (18).

This study tried to investigate the α4, αv, β1 and β3 integrin expression during mouse embryo implantation in the PCOS experimental model. Estrogen and progesterone are necessary for implantation of the blastocyst, and the preimplantation estrogen surge is essential for implantation in mice. It showed that these hormones change with variable patterns in PCOS induced-mice (19). It can be a reason for different integrin expressions in PCOS and normal mice. The surge of estrogen occurs just before implantation, and it prevents attachment of the blastocyst, which stays in a state of diapauses (20). It is identified that in infertile patients with PCOS, a huge percentage of miscarriages occur, spontaneously. In some studies, it is shown that some of these reasons are due to hormonal imbalance such as LH (21). 

Therefore, we have utilized this system to study hormonal modulation of α4, β1, αv, β3 integrin expression in mouse blastocysts during implantation and, we hypothesized that the modulation of α4, β1, αv, β3 integrins by estrogen and progesterone has a direct role in the process of implantation, and that it could be one of the many critical factors facilitating embryo implantation at the fetomaternal interface. We further compared the hormonal modulation, α4, β1, and αv, β3 integrin gene and protein expression on blastocysts of PCOS induced-mice with blastocysts of normal groups. Understanding mechanism and the severity of expression of this marker in this illness can be helpful in making decisions for patients who refer to infertility centers.

## Materials and methods

**Preparation** **of** **PCOS** **animal** **models**

Based on previous studies, thirty mature female mice were randomly allocated to estradiol valerate (EV)-induced PCOS, sham and control groups as a randomized, prospective, controlled study (22). These animals were kept in the experimental animal culture center of the cancer research center of Imam Khomeini hospital. During the study, the animals were kept in standard situation (12:12) and 23±2^o^C.

All the procedures were conducted according to the guidelines for the care and used the Islamic Azad University of Yazd animals’ laboratory. The EV-treated mice were injected estradiol valerate (40 mg/kg body weight) by intramuscular injection [IM] (23). Sham group was injected 100 µl of olive oil. All the groups were evaluated 60 days after the injection.

**Reagents** **and** **culture** **media**

All reagents were purchased from Sigma Aldrich (Germany) except pregnant mare serum gonadotropin (PMSG, Folligon, Intervent, Australia) and human chorionic gonadotropin hormone (HCG, Sereno, Switzerland). For isolation and culture of embryos, T6 medium supplemented with 5 mg/ml BSA was used.

**Body** **weight** **measurement**

During 8 weeks after injection, the body weight of mice was accessed weekly. 

**Measuring** **circulating** **gonadal** **hormones**

Blood samples were collected by heart puncture and serum was isolated by centrifugation (2000 rounds per minute/10 minutes) in 3 mice of each group, 8 weeks after PCOS induction. To determine the effectiveness of estradiol valerate, serum 17-β estradiol and progesterone concentrations were determined by RIA method (a very sensitive in vitro assay technique used to measure concentrations of hormone levels) in Vali-e-asr hospital lab in the Imam Khomeini hospital.

**Histopathological** **studies**

Ovaries of the control, sham and EV-treated mice were removed after the treatment period (n: 3 mice), cleaned from adherent fat and connective tissue, and fixed in 10% formaldehyde buffer for at least 24 hours. Ovaries sections 5-6 μm were routinely processed by standard histological techniques, then ovaries were stained with hematoxylin- eosin stain as described by Guyer, and examined by the light microscope (Olympus BX51, Germany) to assess histopathological changes between, control, sham and experimental animals (24).

**Blastocyst** **collection**

Sixty days after PCO induction, each group was hyper stimulated by 7 IU PMSG and after 48hrs, 7 IU HCG was injected, sixty days after PCO induction. Vaginal plaque was checked, and mice were investigated 5 days after the test. The pregnant mice were sacrificed by cervical dislocation on the morning of 5th day of pregnancy. Embryos were collected by flushing either oviducts with T6 medium (Royan Institute, Iran) supplemented with 5 mg/ml BSA, as what Hou *et* *al* done (20). The numbers of surviving and degenerated embryos were recorded in each group of study using an inverted microscope. The expanded blastocyst with normal morphology was collected and considered for molecular and immunohistochemistry analyses.

**Evaluation** **of** **integrin** **expression** **by** **Real-time** **PCR**

The blastocysts derived from each group (n= 75) were collected and pooled separately (25 embryos for each replicate of experiments). The RNA was isolated from the mice blastocysts using the RN easy Mini Kit (Qiagen, Germany), according to the manufacturer’s instructions. In order to remove genomic contamination, RNA was treated with DNase I, using a kit (EN0521; Fermentas) based on the protocol described by the manufacturers. Concentrations of RNA were determined using a UV spectrophotometer (Eppendorff, Germany). The cDNAs were synthesized from 500 ng DNase-treated RNA samples with a Revert Aid TM first strand cDNA synthesis kit (K1622; Fermentas, Germany) using oligo (dT) primers. For PCR reactions, primers were designed by NCBI site and gene runner software, and synthesized by Cinnagen Company (Table I). 

PCRs were done using Master Mix and SYBR Green I in an Applied Biosystems, Step One TM (Applied Biosystems, USA). The PCR program started with an initial melting cycle, 5 minutes at 95^o^C, to activate the polymerase and followed by 40 cycles as follows: a melting step (30 sec at 95^o^C), an annealing step (30 sec at different temperatures according to table 1) and an extension step (30 sec at 72^o^C). After completing the PCR run, the quality of the reactions was confirmed by melting curve analyses. 

Efficiency was determined for each gene using a standard curve (the logarithmic dilution series of endometer cDNA). For each sample, the reference gene (β-actin) and the target genes were amplified in the same run. Reference genes were approximately equal. The relative quantification of target genes was normalized to a reference gene and determined using the Pfaffl method (25).

**Evaluation** **of** **integrin** **molecules** **by** **Immunohistochemistry**

The blastocysts collected from each group were put on poly-l-lysine coated slides and treated with acid Tyrode's solution (Sigma Aldrich, Germany) for removing the zona pellucida. They were then fixed in 4% paraformaldehyde for 20 minutes and permeabilized by 0.3% Triton-X100 for 30 min. Specific binding was blocked with 10% normal goat serum in PBS. We used antibodies specific for αv (1:100; Chemicon, UK), α4 (1:100; Abcam, UK), β1 (1:250; Abcam, UK) and β3 (1:250; Abcam, UK) diluted in PBS at 4^o^C overnight. The FITC-conjugated secondary rabbit antibodies (Abcam, UK) diluted in PBS were applied for 2 hours. For nuclear staining, 5 μg/ml propidium iodide was used. Images were captured using a Zeiss LSM 5 fluorescent Microscope. All of methods for immunohistochemistry were down according to the manufacturer’s instructions.

**Statistical** **analysis**

All of data in this study have been presented as the mean±SD. The results of body weight, sera estradiol and progesterone and real-time PCR were analyzed by One-way repeated measures analysis of variance (ANOVA) between the groups. P<0.05 were considered statistically significant and followed by the Tukey post hoc test multiple. 

## Results

**Figure 1:**
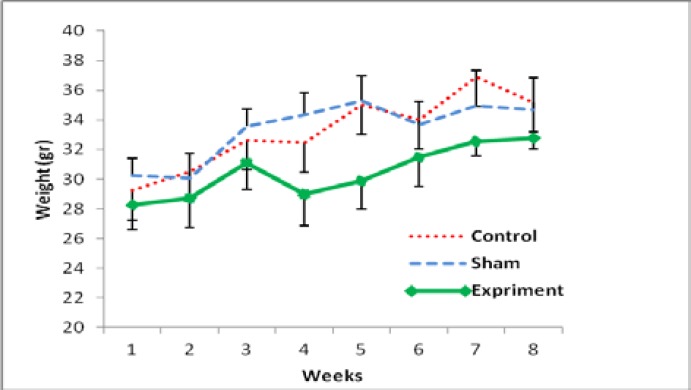
Mean of body weight changes in all experimental groups after 8 weeks of PCOS induction. All groups had a significant increase in the end of study compared to the early week

The results showed that there was an increasing level of weight gaining in all groups. According to the Figure 1, there was not any significant difference between 3 experimental groups after 2 months. Anyway among all groups, there was a significant difference between the first days of study and the last weeks (Figure 1). 

The hormonal assessment showed that the content of 17-β estradiol in sera of control, sham and PCOS groups was 49.14±3.8, 41.17±4.1 and 133.11±5.6 pg/ml and that of progesterone, level was 28±2.01, 35.12±1.72, 18.63±2.11 ng/ml, respectively. The level of 17-β estradiol increased significantly in EV-induced mice compared to the control and sham mice (p<0.035). Also, the level of progesterone decreased significantly in EV-induced mice compared to the other groups' (p<0.048). 

**Figure 2:**
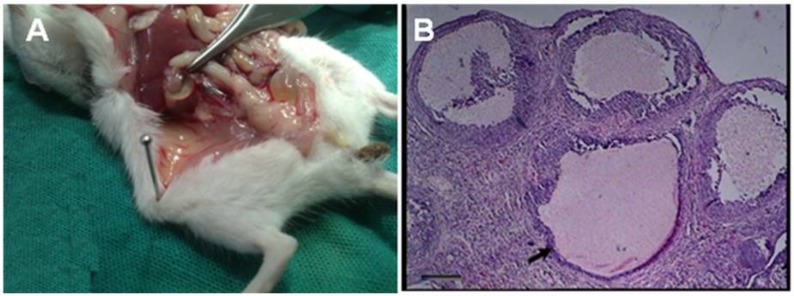
In the ovarian tissue, A; the cysts were mainly appeared by a single intramuscular dose of estradiol valerate, 40 mg/kg (H&E). B; ovarian tissue in control group

Results of PCOS induction showed that a significant difference (p≤0.048) between the amounts of blood serum progesterone in the experimental group compared to other groups, while there wasn't a great difference between the amounts of estrogen in other groups. In the study of tissue sections, the presence of many cysts in ovarian tissue in PCOS induced groups was confirmed (Figure 2). The results indicated that the administration of 40 mg/kg estradiol valerate could be helpful in the induction of PCOS.

Evaluation of molecular part in the amount of αv, β3, β1 and α4 gene expression on blastocyst surface showed that there was a great difference in gene expression β1, β3 and αv between groups. Based on our finding, the ratio of genes' expressions αv, β3, β1 and α4 in PCOS to control group was 0.479±0.01, 0.5±0.001, 2.7±0.4 and 1.023±0.2 respectively. Also, the amount of genes' expressions ratio in sham to control group at the same integrins was 0.935±0.07, 1.02±0.2, 1.3±0.2 and 0.912±0.09 respectively.

**Figure 3:**
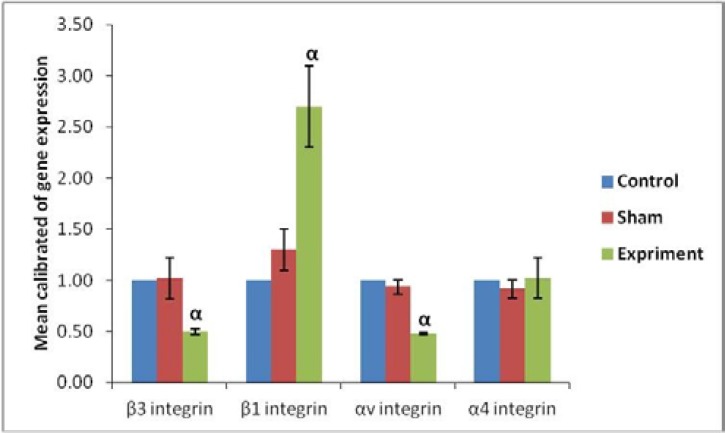
Real Time-PCR analysis. mRNA levels were normalized with respect to β-actin, chosen as an internal control and calibrated to control group. Profile of Integrin genes' expression on the blastocyst surface after using a single intramuscular dose of estradiol valerate, 40 mg/kg. Histograms show mean expression values (±SD; p<0.05). α: significant difference with other groups in the same genes.

The results showed that β1 expressions in the blastocysts of PCOS-induced mice were significantly (p≤0.05) increased whereas that of β3 and αv expression was significantly (p≤0.05) decreased compared to other groups; however, the expression observed in the control and sham groups was not significant in none of the integrin genes (Figure 3).

**Figure 4:**
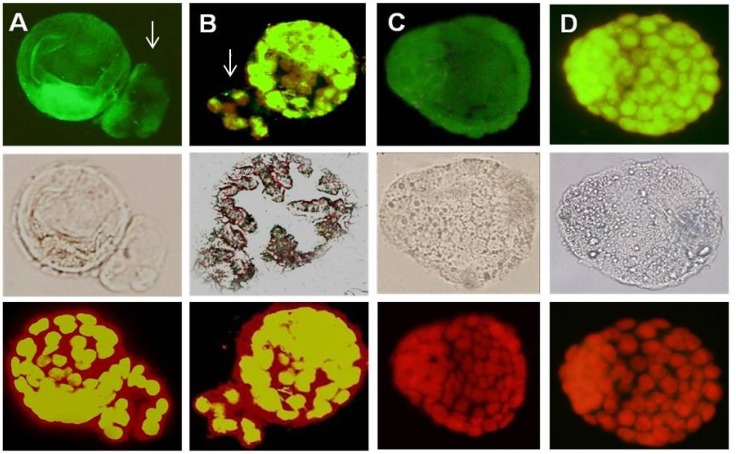
Immunostaining reactivity of normal blastocyst mice

**Figure 5:**
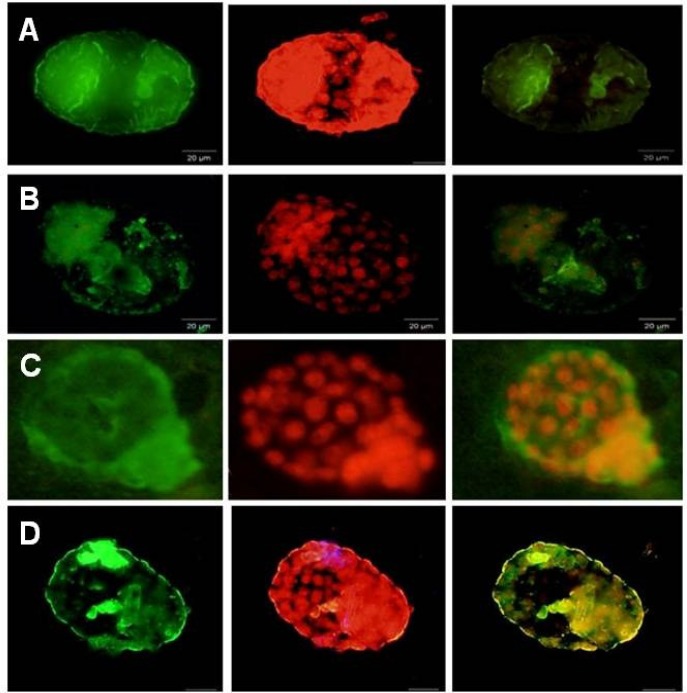
Immunostaining reactivity of PCOs blastocyst mice. A; *β1 integrin* expression, B; *αV integrin* expression, C; *α4 integrin *expression, D; *β3 integrin *expression

The αv, α4, β1 and β3 integrins were detected in the blastocyst surface of all groups during the implantation window (Figure 4, 5 A-D). β1 subunits were most intense in the PCOS group compared to control (Figure 5 A) but αv and β3 subunits showed a different pattern compared to α4 and β1 subunits in PCOS induce-mice group (Figure 4 A, B, 5 B, D). αv and β3 subunits were detected poor in the blastocyst surface of PCOS-induced mice during of the implantation window. All of integrin molecules expressions were expressed on the surface of the hatched embryos, but their expressions were restricted in the surface of the hatched embryos in PCOS-induced mice.

**Table I:** Primer Used for real- time PCR

**Genes**	**Primer sequence**	**GenBank code**	**Tm (** ^o^ **C)**
β1 integrin	FOR: 5′- TGCCTACAACTCTCTTTCTTC-3′	NM_010578.2	58
	REV: 5′- TGGTTTCAGACTCCTTATTTG-3′		
β-actin	FOR: 5΄- TCCCTGGAGAAGAGCTACG-3΄	NM_001101	66.6
	REV: 5΄- GTAGTTTCGTGGATGCCACA-3΄		
β3 integrin	FOR: 5΄-TGGAAGAGCCTGAGTGTC-3΄	NM_016780	60
	REV: 5΄-CGGTAGGTGATATTGGTGAAG-3΄		
αV integrin	FOR: 5΄- GGAACAACGAAGCCTTAG-3΄	NM_008402	55
	REV: 5΄- GTATCCATCTCTGACTGC-3΄		
α4 integrin	FOR: 5׳- GAATCTCCTCCACCTACTCACAG -3׳	NM_010576	64
	REV: 5׳-CCAACGGCTACATCAACATATCC-3׳		

A; *β3 integrin* expression, B; *αV integrin* expression, C; *α4 integrin *expression, D; *β1 integrin *expression. Arrow: hatched blastocyst.

## Discussion

PCOS induction and estrous cycle fixation by estrogen injection in female mature mice are possible. Therefore, in order to make a close relationship between research and clinical situations, PCOS in mature mice was induced by means of the most common way (estradiol valerate). PCOS symptoms in mice, who have received 40 mg/kg estradiol valerate, were completed after 8 weeks. Beginning steps of PCOS is identified through blood estrogen increase and progesterone decrease. This part of study was shown previously as well (26-28). 

In this model of PCOS, due to the effect of estradiol valerate on the hypophysis-hypothalamus pathway; sexual hormone secretion is impaired, and ovules are affected indirectly too and make cysts. On the basis of previous studies, a change in sexual hormone level due to a PCOS induction after 8-12 weeks reaches to the extent that ovules become completely polycystic (29). 

According to Luza and her colleagues, in 1995 and Brawer in 1978, by estradiol valerate injection, hypothalamus-hypophysis pathway is impaired. This impairment may cause a change in secretion pattern of hypothalamus and then hypophysis. Therefore, it can affect the sexual hormone levels in blood serum (30-31). We have demonstrated; for the first time, the expression of several integrins molecules on blastocyst's surfaces of PCOS-induced mice, during implantation window using real-time RT-PCR in combination with immunocytochemistry techniques. 

Our observations, obtained by two techniques, demonstrated that there was a significant difference in expression of integrins on blastocysts surface of PCOS mice during the implantation window. Other pervious results showed that all examined integrins were expressed on the metestrus phase at the mRNA and protein levels, and they were not detected on the other phases in the uterus of mice (32). We concluded that the expression of these molecules on the surface of the blastocyst was controlled by progesterone whereas the estrogen had minor effect in this regard. It is more difficult to realize what regulates the expression of integrin molecules during the implantation time. The expressions of epithelial αv, β3 integrin 6-8 days after ovulation, since the timing of expression take places well after the increase in serum progesterone in pig (33). 

Widra *et* *al* have explained that estrogen down-regulated αv, β3 expression, but no effect was detected for α4 or β1 integrin subunit (34). In contrast to our description, Basak et al showed the down-regulation of α4 integrin expression by progesterone and its upregulation by estradiol in the mouse blastocysts and the endometrium by a delayed-implantation mouse model (35). Moreover, some in vivo and in vitro researches have shown that the estrogen and progesterone decrease the expression of αv, β3 integrin heterodimers or its expression not influenced by sex steroids in vivo (36-39). 

Fayazi *et* *al* study, showed a significant decrease in the expression of αv, β1, and β3 integrins at the mRNA levels of blastocysts derived from the ovarian stimulated mice compared to the control group. Their data confirmed that the concentration of the 17-β estradiol and progesterone in stimulated mice was ten and two folds more than the control mice, respectively. They concluded that the expressions of the integrin molecules were controlled by ovarian hormones, thus their imbalance in stimulated subjects affected to their integrin molecule expression (18). In this regard, Lin *et* *al* demonstrated no correlation between the serum levels of steroid hormones and the mRNA expression of αv and β3 integrins, thus they concluded that steroid hormones might cooperate a minor role in the regulation of αv and β3 integrin expression (40). 

These controversially outcomes may be caused by the various types of integrin molecules, which were investigated by different protocols and experimental design or these different results related to several types of species, which were used, because there were some varieties in the implantation process, including invasive and non-invasive types. Integrins molecules are one of the common receptors on the blastocyst surface in all mammals, and their expression patterns are in cyclic (18). In this study, expression pattern in αv, β3, β1 and α4 on the blastocyst surface in PCOS induced-mice were evaluated.

The comparison of mRNA expression ratio of different integrins and housekeeping genes revealed that the α4 subunit in the experimental group does not have the prominent expression than other molecules, and its mRNA was detected similar to other groups. This observation reflected the importance of αv, β3 and β1 more than the α4 molecules in implantation of the embryo. The β3 subunits almost co-expressed with αv on the blastocyst surface. 

These observations reflected the roles and importance of different types of integrins heterodimers including α4, β3 α4, β1, αv, β1, αv, β3 in different site of endometrium not only for creation of contact between the embryo and endometrium but also for embryo penetration during implantation period. In this regard, Basak *et* *al* explained the expression of α4 integrin on the basement membrane of the uterus and proposed that it might facilitate in attachment of the blastocyst to the uterine lumen. 

Additionally, they have blocked the α4 integrin by monoclonal antibodies in the uterus of pregnant mice on the day of implantation and explained the implantation failure in both normal as well as delayed implantation mice (35). But in this study, we showed the α4 expression does not have any significant difference compared with Basak *et* *al* study. Regulating of these markers and their role in implantation molecule mechanism in human is unknown. Therefore, according to other studies, it was shown that hormonal changes due to PCOS can be affected on gene appearance and other proteins involved in endometrial and embryo linking.

## Conclusion

According to the results obtained from this study, in mice with PCOS, αv and β3 integrin expressions on the blastocyst surface are significantly lower than normal mice. It seems that in PCOS induced-mice, this pattern has changed and the results have a great effect on spontaneous miscarriage. Therefore, there must be a great attention toward this problem in patients who are involved. 

## References

[1] Speroff L, Fritz MA (2011). Cronic anovulation and the polycystic ovary syndrome. Clinical gynecoligic endocrinology and infertility.

[2] Qiao J, Wang L, Li R, Zhang X (2008). Microarray evaluation of endometrial receptivity in chinese women with polycystic ovary syndrome. Reprod Biomed Online.

[3] Cakmak H, Taylor HS (2011). Human implantation failure: molecular mechanisms and clinical treatment. Hum Reprod Update.

[4] Donaghay M, Lessey BA (2007). Uterine receptivity: alterations associated with benign gynecological disease. Semin Reprod Med.

[5] Lopes IM, Baracat MC, Simões Mde J, Simões RS, Baracat EC, Soares Jr JM (2011). Endometrium in women with polycystic ovary syndrome during the window of implantation. Rev Assoc Med Bras.

[6] Paria BC, Huet-Hudson YM, Dey SK (1993). Blastocyst’s state of activity determines ‘‘window’’ of implantation in the receptive mouse uterus. Proc Natl Acad Sci USA.

[7] Aplin J (1997). Adhesion molecules in implantation. Rev Reprod.

[8] Harvey MB, Leco KJ, Arcellana-Panlilio MY, Zhang X, Edwards GR, Schultz GA (1995). Roles of growth factors during peri- implantation development. Mol Hum Reprod.

[9] Saito S (2000). Cytokine network at the fetomaternal interface. J Reprod Immunol.

[10] Chakraborty I, Das SK, Dey SK (1995). Differential expression of vascular endothelial growth factor and its receptor mRNAs in the mouse uterus around the time of implantation. J Endocrinol.

[11] Hynes RO (1992). Integrins: versatility, modulation and signaling in cell adhesion. Cell.

[12] Damsky C, Sutherland A, Fisher S (1993). Extracellular matrix 5: adhesive interactions in early mammalian embryogenesis, implantation and placentation. FASEB J.

[13] Yoshimura Y (1997). Integrin: expression, modulation and signaling in fertilization, embryogenesis and implantation. Kieo J Med.

[14] Sutherland AE, Calarco PG, Damsky CH (1993). Developmental regulation of integrin expression at the time of implantation in the mouse embryo. Development.

[15] Aplin J (1997). Adhesion molecules in implantation. Rev Reprod.

[16] Reddy KV, Meherji PK (1999). Integrin cell adhesion molecules in endometrium of fertile and infertile women throughout menstrual cycle. Indian J Exp Biol.

[17] Gonzalez RR, Palomino A, Boric A, Vega M, Devoto L (1999). A quantitative evaluation of a1, a4, aV and b3 endometrial integrins of fertile and unexplained infertile women during the menstrual cycle. A flow cytometric appraisal. Hum Reprod.

[18] Fayazi M, Beigi Brojeni M, Salehnia M, Khansarinejad B (2014). Ovarian stimulation by Exogenous Gonadotropin Decreases the Implantation Rate and Expression of Mouse Blastocysts Integrins. Iran Biomed J.

[19] Wood JR, Dumesic DA, Abbott DH, Strauss JF 3rd (2007). Molecular Abnormalities in Oocytes from Women with Polycystic Ovary Syndrome Revealed by Microarray Analysis. J Clin Endocrinol Metab.

[20] Hou Q, Paria BC, Mui C, Dey SK, Gorsky J (1996). Immunolocalization of estrogen receptor protein in the mouse blastocyst during normal and delayed implantation. Proc Natl Acad Sci USA.

[21] Norman RJ, Dewailly D, Legro RS, Hickey TE (2007). Polycystic ovary syndrome. Lancet.

[22] Dikmen A, Ergenoglu AM, Yeniel AO, Dilsiz OY, Ercan G, Yilmaz H (2012). Evaluation of glycemic and oxidative/antioxidative status in the estradiol valerate-induced PCOS model of rats. Eur J Obstet Gynecol Reprod Biol.

[23] Mahood RAH (2012). Effects of Pimpinella anisum oil Extract on Some Biochemical Parameters in Mice experimentally induced for human Polycystic Ovary Syndrome. J Biotec Res Cent.

[24] Guyer M (1993). Animal microbiology.

[25] Pfaffl MW (2001). A new mathematical model for relative quantification in real-time RT-PCR. Nucleic Acids Res.

[26] Stener-Victorin E, Kobayashi R, Watanabe O, Lundeberg T, Kurosawa M (2004). Effect of electro-acupuncture stimulation of different frequencies and intensities on ovarian blood flow in anaesthetized rats with steroid-induced polycystic ovaries. Reprod Biol Endocrinol.

[27] Schulster A, Farookhi R, BrawerJ R (1984). Polycystic ovarian condition in Estradiol Valerate-treated rats: spontaneous changes in characteristic endocrine features. Biol Reprod.

[28] Shirwalker H, Deepak N M, Anurupa M (2007). Exposure of adult rats to estradiol valerate induces ovarian cyst with early senescence of follicles. Mol Cell Endocrinol.

[29] Stener-Victorin E, Lundeberg T, Waldenstrom U, Manni L, Aloe L, Gunnarsson S (2000). Effects of Electro-Acupuncture on Nerve Growth Factor and Ovarian Morphology in Rats with Experimentally Induced Polycystic Ovaries. Biol Reprod.

[30] Luza SM, LizamA L, Burgos RA, Lara HE (1995). Hypothalamic Changes in norepinephrine Release in Rats with EstradiolValerate-Induced Polycystic Ovaries. Biol Reprod.

[31] Brawer JR, Naftolin F, Martin J, Sonnenschein C (1978). Effects of a single injection of estradiol valerate on the hypothalamic arcuate nucleus and on reproductive function in the female rat. Endocrinology.

[32] Peyghambari F, Salehnia M, Forouzandeh Moghadam M, Rezazadeh Valujerdi M, Hajizadeh E (2010). The correlation between the endometrial integrins and osteopontin expression with pinopodes development in ovariectomized mice in response to exogenous steroids hormones. Iran Biomed J.

[33] Lessey BA, Damjanovich L, Coutifaris C, Castelbaum A, Albelda SM, Buck CA (1992). Integrin adhesion molecules in the human endometrium Correlation with the normal and abnormal menstrual cycle.. J Clin Invest.

[34] Widra EA, Weeraratna A, Stepp MA, Stillman RJ, Patierno SR (1997). Modulation of implantation-associated integrin expression but not uteroglobin by steroid hormones in an endometrial cell line. Mol Hum Reprod.

[35] Basak S, Dhar R, Das C (2002). Steroids modulate the expression of alpha4 integrin in mouse blastocysts and uterus during implantation. Biol Reprod.

[36] Kimmins S, MacLaren LA (1999). Cyclic Modulation of integrin expression in bovine endometrium. Biol Reprod.

[37] Lessey BA, Arnold JT (1998). Paracrine signaling in the endometrium: integrins and the establishment of uterine receptivity. J Reprod Immunol.

[38] Apparao KB, Murray MJ, Fritz MA, Meyer WR, Chambers AF, Truong PR (2001). Osteopontin and its receptor alphavbeta (3) integrin are coexpressed in the human endometrium during the menstrual cycle but regulated differentially. J Clin Endocrinol Metab.

[39] Bowen JA, Bazer FW, Burghardt RC (1996). Spatial and temporal analyses of integrin and Muc-1 expression in porcine uterine epithelium and trophectoderm in vivo. Biol Reprod.

[40] Lin H, Wang X, Liu G, Fu J, Wang A (2007). Expression of alpha V and beta 3 integrin subunits during implantation in pig. Mol Reprod Dev.

